# Exploring the mediating role of self-efficacy beliefs among EFL university language learners: The relationship of social support with academic enthusiasm and academic vitality

**DOI:** 10.1016/j.heliyon.2024.e33253

**Published:** 2024-06-18

**Authors:** Qin Luo, Roya Ahmadi, Siros Izadpanah

**Affiliations:** a English Department, School of Foreign Languages, Beijing Institute of Technology, Beijing, 102401, China; b Department of English Language Teaching, Zanjan Branch, Islamic Azad University, Zanjan, Iran

**Keywords:** Academic enthusiasm, Academic vitality, Educational environment, English language learners, Mediating role, Self-efficacy beliefs, Social support

## Abstract

This study explores the intricate relationship between social support, academic enthusiasm, and academic vitality among English language learners (ELLs) in 2023, with a specific emphasis on the intermediary role of self-efficacy beliefs. Despite the existing body of literature, there has been a notable dearth of discussions concerning the influence of social support on academic enthusiasm and vitality. In 2023, the educational landscape is evolving rapidly, demanding a deeper understanding of the factors that drive student motivation and academic engagement. This study addresses this gap by investigating the role of social support and self-efficacy beliefs in shaping academic enthusiasm and vitality among ELLs in this contemporary educational context. Through a quantitative research approach, this study engaged a cohort of 242 ELLs from Zanjan University, encompassing both genders. Data were diligently collected through the administration of four distinct questionnaires by using multiple-stage cluster sampling. To unearth insights into the complex relationships under scrutiny, data analyses were meticulously conducted using SPSS 25 and AMOS 24. The consequential findings underscore the paramount significance of cultivating a supportive educational milieu that effectively bolsters self-efficacy beliefs. This nurturing environment, in turn, begets heightened academic enthusiasm and vitality among ELLs. The implications of these findings are manifold, offering universities a valuable toolkit to forge strategies and interventions aimed at fostering not only social support but also the crucial underpinning of self-efficacy beliefs. By doing so, these institutions can effectively nurture the academic enthusiasm and vitality of ELLs, thereby enhancing their educational experience and outcomes.

## 1 Introduction

The realm of education stands as a dynamic crucible where students embark on a journey of growth, development, and adaptation. In the pursuit of academic excellence, EFL learners navigate an intricate landscape, facing a myriad of challenges that necessitate a nuanced understanding of adaptive strategies [1]. To fortify the educational journey in the EFL context, it is crucial to fathom the profound impact of various determinants on learners' academic enthusiasm and vitality.

Social support is a multifaceted construct, encompassing emotional nurturance, practical assistance, and a profound sense of belonging within a supportive network ([2]; Falcon et al. [3]). It extends beyond the confines of emotional solace to include tangible resources and interactions that EFL individuals receive to confront life's trials [4]. Rooted in the broader notion of social capital, social support emanates from family, friends, and acquaintances, bridging dimensions like instrumental aid, informational guidance, and emotional sustenance. Within the realm of EFL education, the role of social support is pivotal, emerging as an influential factor that significantly impacts EFL learners [4,5].

However, while the literature acknowledges the importance of social support, the nexus between this construct and two crucial facets of the academic journey in the EFL context, academic enthusiasm, and vitality, warrants deeper exploration. Academic enthusiasm, characterized by fervor, dedication, and an unwavering sense of absorption, stands as a potent antidote to academic burnout among EFL learners [6]. Academic vitality, on the other hand, embodies EFL learners' constructive and adaptive responses to educational challenges, featuring heightened motivation and a robust sense of self-efficacy (Dehghanizadeh et al.[7] [8]; Wang & Sheikh [9]).

In the ever-evolving landscape of EFL education in 2023, as pedagogical paradigms shift and technological advancements reshape the EFL learning experience, understanding the intricate dynamics of social support's influence on academic enthusiasm and vitality among EFL learners becomes increasingly relevant. However, existing discourse often falls short in illuminating the mediating factors at play within the specific context of EFL education.

This research aims to address this gap by shedding light on the mediating role of “self-efficacy beliefs” within the EFL context. Anchored in Bandura's social cognitive theory, self-efficacy beliefs shape human motivation, behavior, and cognitive processes [10,11]. These beliefs underpin human actions, fuel self-regulation, and guide individuals in their response to challenges [12]. In the realm of EFL education, self-efficacy beliefs are central to influencing EFL learners' effort levels and persistence ([13]؛ Zimmerman et al. [14]). However, despite the wealth of existing literature, the intricate mediating factors intrinsic to academic enthusiasm and vitality among EFL learners remain understudied.

This research aspires to bridge this gap by elucidating the mediating role of self-efficacy beliefs in the relationship between social support and academic enthusiasm and vitality within the specific context of EFL education. In an era where EFL education continually evolves, presenting new challenges and opportunities, this research assumes paramount significance for EFL educators and institutions. By uncovering the dynamics of social support, self-efficacy beliefs, and academic outcomes within the EFL context, this study contributes to a deeper understanding of the educational landscape for EFL learners. The findings empower EFL educational institutions to create environments fostering not only social support but also the vital underpinning of self-efficacy beliefs, thereby enhancing the academic journey of EFL learners.

## 2 Review of literature

English as a Foreign Language (EFL) university learners encounter various challenges in their language acquisition journey, where social support plays a crucial role in their academic success and well-being [15]. Within the context of EFL education, academic enthusiasm, and vitality are pivotal for learners' engagement and motivation [16].

The relationship between social support and academic outcomes in EFL education has garnered increasing attention [17]. Social support, including emotional, instrumental, and informational assistance, contributes significantly to learners' academic experiences [18]. Academic enthusiasm, characterized by cognitive engagement and purposeful learning, is fundamental in the EFL context [19]. Similarly, academic vitality, reflecting learners' motivation and resilience, is essential for successful language acquisition [15].

Self-efficacy beliefs, rooted in Bandura's social cognitive theory, are influential in shaping learners' motivation and persistence. Previous research suggests that self-efficacy beliefs mediate the relationship between social support and academic outcomes among university language learners [20]. By examining the interplay between social support, academic enthusiasm, and academic vitality, this study seeks to elucidate how self-efficacy beliefs function as a mediator in this relationship.

Understanding these dynamics is crucial for optimizing support systems and enhancing the learning experiences of EFL university language learners. Through this investigation, we aim to contribute to the theoretical understanding of the mechanisms underlying academic enthusiasm and vitality in EFL education.

### 2.1 Social support in EFL education

In the realm of English as a Foreign Language (EFL) education, social support plays a pivotal role in facilitating the language acquisition journey of learners navigating unfamiliar linguistic and cultural terrains [21]. Originating from Cohen's developmental psychology framework, the concept of social support extends beyond childhood development to become a cornerstone for EFL learners overcoming language barriers and adapting to academic environments [22].

Social support within EFL education encompasses nurturing relationships within classrooms and broader networks of peers, teachers, and online communities ([21]. These support systems serve as secure bases from which EFL learners draw strength, seek guidance, and find belonging, contributing to academic success and language proficiency.

### 2.2 Academic enthusiasm and vitality in EFL learning

Academic enthusiasm is gaining recognition in educational research, particularly within the EFL context, characterized by cognitive engagement, persistence, and purposeful learning experiences [23]. EFL learners exhibiting academic enthusiasm demonstrate heightened cognitive performance and active participation in language learning activities, fostering positive psychological variables [24].

The synergy between social support and academic enthusiasm is pronounced in EFL education [25]. Perceived social support from peers, instructors, and family members propels EFL learners into a state of heightened academic enthusiasm, enhancing engagement and positive emotional states [26].

### 2.3 Social support: an evolving concept

In the context of English as a Foreign Language (EFL) education, the concept of social support takes on a particular significance. EFL learners, often navigating unfamiliar linguistic and cultural terrains, rely heavily on social support networks to facilitate their language acquisition journey (Falcon et al. [3]). While the origins of social support theory may be traced back to Cohen's work in developmental psychology, its applicability extends far beyond childhood development [27]. Within the EFL context, social support becomes a cornerstone for learners seeking to overcome language barriers, adapt to new academic environments, and flourish in their language-learning endeavors.

Social support in the realm of EFL education encompasses not only the nurturing relationships within the classroom but also extends to the broader network of peers, teachers, and even online communities [8,28]. These support systems serve as secure bases from which EFL learners can draw strength, seek guidance, and rely upon as they navigate the intricacies of language acquisition. Within this context, social support becomes a multifaceted construct, encompassing emotional encouragement, practical assistance, and a sense of belonging within the EFL learning community.

As the field of EFL education evolves, embracing innovative pedagogical approaches and technological advancements, the role of social support remains paramount. EFL learners' academic success and language proficiency are intrinsically linked to the support structures available to them. Thus, understanding the dynamics of social support within the EFL context is essential for educators and researchers alike.

### 2.4 Academic enthusiasm

In the realm of education, academic enthusiasm, although a relatively recent construct, has been gaining substantial recognition within educational research [29]. Recent studies have delineated academic enthusiasm as a multifaceted concept within the EFL context, blending cognitive performance, unwavering persistence, active engagement in collaborative learning experiences, and a resolute sense of purpose ([30]; Reynolds & Walberg [31]). This construct is further elucidated as a positive emotional-motivational state specifically tied to academic endeavors, comprising three vital facets: energy, dedication, and passion.

In the context of EFL learning, academic enthusiasm emerges as a cornerstone that underpins various aspects of the educational journey [32]. EFL learners who exhibit academic enthusiasm not only display heightened cognitive performance and a steadfast commitment to their language studies but also actively participate in collaborative language learning activities with purpose and fervor [33]. This enthusiasm becomes a catalyst for transformative efforts within the field of EFL education, exerting a profound influence on an array of psychological variables. These variables encompass EFL learners' beliefs in their language acquisition abilities, their ability to set and pursue language learning goals, and the quality of their social connections within the EFL learning community.

### 2.5 Social support and academic enthusiasm

In the realm of EFL education, the synergy between social support and academic enthusiasm becomes particularly pronounced and influential ([13]; Wu et al. [34]). Social support, encompassing emotional, instrumental, and informational assistance from various social networks, is intricately interwoven with the academic experiences of EFL learners [8]. Within this context, EFL learners who perceive a heightened level of social support from peers, instructors, and family members often find themselves propelled into a state of heightened academic enthusiasm [35].

Social support, in the EFL context, plays a multifaceted role in nurturing EFL learners' language acquisition journeys. It fosters a profound sense of belonging within the EFL learning community, offering emotional encouragement, guidance, and validation that are essential for the language learning process (Alipour Katigari et al. [36]). This supportive network, composed of peers, instructors, and family members, contributes significantly to shaping not only positive emotional states but also cognitive behaviors related to academic pursuits among EFL learners.

EFL learners who receive substantial social support within their language learning endeavors are more likely to exhibit not only an unwavering passion for learning but also active engagement in a range of academic activities [5]. This heightened level of engagement, fueled by the support network, becomes a driving force behind EFL learners' motivation to persist in their language studies. As we delve deeper into the subsequent sections, we explore the intricate dynamics of social support and its profound impact on the academic enthusiasm of EFL learners, shedding light on the multifaceted relationships that shape the EFL educational landscape.

### 2.6 Academic vitality

In the context of EFL education, the concept of academic vitality, characterized by a vibrant sense of energy, enthusiasm, and self-motivation, takes on unique dimensions [37]. Academic vitality within the EFL realm is deeply intertwined with the broader notion of vitality, which is closely linked to mental well-being and the essence of life itself [38,39]. EFL learners who exhibit academic vitality are distinguished by their high levels of motivation, a strong sense of self-efficacy, and adaptive responses to the challenges inherent in language acquisition. This vitality is inherently connected to the principles of self-determination theory (Deci et al. [40]) within the EFL learning context. This theory underscores the innate human desire for learning, which can flourish with the appropriate environmental support.

Within the EFL educational landscape, the principles of self-determination theory are particularly relevant, emphasizing both internal and external motivations [41]. EFL learners who experience academic vitality draw strength from a sense of autonomy in their language learning journey, recognizing their worth, and forging meaningful connections with others within the EFL community. This sense of vitality and motivation among EFL learners is instrumental in driving autonomously initiated behaviors and contributes significantly to their success in mastering a new language.

### 2.7 Social support fosters academic vitality

Within the context of EFL education, the role of social support in fostering academic vitality takes on paramount importance [37]. EFL learners, often navigating the intricate journey of language acquisition, greatly benefit from a supportive social network that contributes significantly to the development of their academic vitality.

In the EFL context, academic vitality is marked by traits such as unwavering persistence, resilience in the face of challenges, and a positive attitude towards language learning [42]. These attributes are crucial for EFL learners as they encounter academic hurdles associated with language acquisition. Social support plays a pivotal role as a catalyst for these attributes to flourish among EFL learners.

Social support acts as a protective shield against stress and anxiety, creating an environment conducive to the cultivation of academic vitality [43]. It empowers EFL learners to navigate language-related difficulties with enthusiasm and determination [44]. This support network not only enables learners to maintain their motivation but also equips them with adaptive coping strategies, essential for surmounting the unique challenges posed by learning a new language.

### 2.8 Self-efficacy beliefs

In the unique context of EFL education, self-efficacy beliefs, deeply rooted in Bandura's influential social cognitive theory [45], hold a pivotal position in shaping motivation, well-being, and academic achievement. These beliefs revolve around an individual's confidence in their ability to successfully perform specific language-related tasks and achieve desired language learning outcomes.

Within the EFL context, self-efficacy beliefs serve as guiding forces that significantly influence human behavior. These beliefs impact the choices EFL learners make, the effort they invest in their language studies, and their persistence in the face of linguistic challenges [13]. Specifically, self-efficacy beliefs within the EFL context predict language learning performance and play a profound role in determining students' willingness to invest time and effort into their language studies.

EFL learners who possess higher levels of self-efficacy beliefs are more likely to embrace linguistic challenges as opportunities for growth. Their confidence in their language learning abilities empowers them to tackle complex language tasks with determination and enthusiasm (Wu et al.[34]). As a result, these learners tend to exhibit enhanced language learning outcomes, mastering EFL more effectively and with greater ease.

### 2.9 Self-efficacy beliefs as mediators

In the context of EFL education, self-efficacy beliefs play a pivotal and mediating role in shaping the landscape of academic enthusiasm and vitality. These beliefs serve as essential links between the influence of social support and the ultimate academic outcomes of EFL learners [13].

EFL learners with high self-efficacy beliefs perceive themselves as capable individuals, even when faced with the unique challenges associated with language learning [10]. This unwavering confidence in their language learning abilities empowers them to navigate linguistic hurdles with determination and resilience. Importantly, research conducted within the EFL context suggests that self-efficacy beliefs act as mediators, connecting the influence of social support to the levels of academic enthusiasm and vitality experienced by university language learners [46].

When EFL learners receive substantial social support, their self-efficacy beliefs are significantly reinforced. This reinforcement, in turn, leads to a noticeable increase in their academic enthusiasm and vitality. Social support networks, encompassing peers, instructors, and family members, contribute to the strengthening of EFL learners' self-efficacy beliefs (Jeong et al. [47]). As these beliefs grow in strength, EFL learners become more motivated, engaged, and resilient in their language learning efforts, ultimately contributing to enhanced academic outcomes.

In summary, this critical review of literature underscores the intricate interplay between social support, academic enthusiasm, academic vitality, and self-efficacy beliefs within the context of university language learners. Understanding these relationships is pivotal for optimizing educational environments and enhancing students' learning experiences. This exploration serves as a foundation for the forthcoming study, shedding light on the mediating role of self-efficacy beliefs in the relationship between social support and academic outcomes. To further enhance the clarity and focus of our research, we now introduce a set of hypotheses that articulate the specific relationships and expectations guiding our investigation.

### 2.10 Hypotheses

1. There is a significant relationship between social support and academic enthusiasm among EFL university language learners.
2. There is a significant relationship between social support and academic vitality among EFL university language learners.
3. There is a significant relationship between self-efficacy beliefs and academic enthusiasm of EFL university language learners.
4. There is a significant relationship between self-efficacy beliefs and the academic vitality of EFL university language learners.
5. Social support has a significant on academic enthusiasm with the mediating role of self-efficacy beliefs in EFL university language learners.
6. Social support has a significant on academic vitality with the mediating role of self-efficacy beliefs in university language learners.

## 3 Method

### 3.1 Design of study

The current research employs a quantitative and correlational descriptive research design, primarily adopting an exploratory research approach. The data collection methodology for this study relies on quantitative techniques, with a specific focus on structured questionnaires designed to elicit numerical responses. The utilization of surveys facilitates the collection of quantitative data, ensuring a rigorous and systematic approach to information gathering. Furthermore, the study incorporates elements of an experimental design due to the random selection of participants, introducing controlled variability to the research.

### 3.2 Participants

The study encompassed a population of 650 ELLs from Zanjan University, Iran. Participant selection employed a random sampling technique. Following Morgan's table for sample size determination (1970), the final sample consisted of 242 ELLs, representing both male and female students. These ELLs had ages ranging from 18 to 55 years and were categorized into three age groups: 18–25 years, 26–30 years, and 45–55 years. It is noteworthy that the majority of participants fell within the 26–30 years age range.

### 3.3 Sampling method

The sampling process involved two stages: First Stage (Convenience Sampling): Initially, 750 students who volunteered for the study were selected using convenience sampling. After administering the Oxford placement test, 650 individuals were identified as having intermediate-level proficiency. Second Stage (Simple Random Sampling): Given the required sample size of 242 individuals for the study, a second stage of sampling was conducted. From the pool of 650 individuals with intermediate-level proficiency, a sample of 242 participants was randomly selected using simple random sampling. This approach allowed for the efficient selection of participants, beginning with a larger group identified through convenience sampling and then narrowing down to a representative sample through random selection based on predetermined criteria.

### 3.4 Instruments

To evaluate the language proficiency level of the participants and ensure a homogeneous group, the Oxford Quick Placement Test (2007) was employed. This test categorizes participants into different proficiency levels as follows:

1-17: Beginner
18-27: Elementary
28-36: Lower-intermediate
37-47: Upper-intermediate
48-55: Advanced
56-60: Very advanced

**Academic Enthusiasm Questionnaire:** Academic enthusiasm in this study was assessed using 15 items derived from Fredericks Blumenfield Paris's (2004) questionnaire. These items are categorized into three subscales: behavioral passion (items 1, 2, 3, and 4), emotional passion (items 5, 6, 7, 8, 9, and 10), and cognitive passion (items 11, 12, 13, 14, and 15). Participants rated their responses on a 5-point scale, ranging from “completely agree” to “completely disagree.” Notably, items 2, 4, and 6 were reverse-scored. The questionnaire's formal validity was assessed by five psychology department professors, who rated its suitability on a 5-point scale from “not at all suitable” (1) to “very suitable” (5). The academic enthusiasm questionnaire, along with its components, received a high rating of appropriateness. The scale's reliability was established in previous studies, with reported Cronbach's alpha coefficients of 0.66 and 0.86 [35].

**Social** Support **Questionnaire:** Social support was measured using 23 items from Wax et al.'s (1986) questionnaire. This questionnaire encompasses three components: family support, friends' support, and support from others. Participants evaluated each item on a 5-point scale, ranging from “very little” (1) to “very much” (5). Certain questions (3, 10, 13, 21, and 22) were reverse scored. The reliability of this questionnaire was examined in various studies, resulting in coefficients of 1.91 and 1.71 in different samples (Ebrahimi Qavam). Shah Bakhsh (2008) calculated an internal reliability coefficient of 1.66 with a group of 311 students from Allameh Tabatabai University, while Khabaz et al. (2019) reported an alpha coefficient of 1.74 for this questionnaire.

**Academic Vitality Questionnaire:** Academic vitality was measured using a 9-item vitality questionnaire developed by Hossein Chari and Dehghanizadeh [48]. The questionnaire employed a 5-point scale ranging from “completely disagree” (1) to “completely agree” (5). The highest possible score for this variable is 45, with the lowest being 9. The validity of this questionnaire was established by examining the correlation of each item with the total score, resulting in correlations ranging from 0.54 to 0.64 (Dehghanizadeh et al. [7]). The reliability of the academic vitality scale was assessed using Cronbach's alpha, with a coefficient of 0.77 reported in their study.

**Self-efficacy Beliefs Questionnaire:** Self-efficacy beliefs were measured using a 17-item questionnaire developed by Sherer et al. [49]. This questionnaire employed a 5-point scale, ranging from “completely disagree” (1) to “completely agree” (5). The highest achievable score for this variable is 85, while the lowest is 17. The validity of this questionnaire was confirmed through its use in previous studies. Barati (1376) employed the questionnaire with third-grade students of Nizam-New High School, assessing reliability through the bisection method. The reliability coefficient was found to be 0.76 using Spearman-Brown with equal length and unequal length, and 0.75 through Guttman's halving method. Additionally, construct validity was confirmed by correlating the scale with self-esteem and self-evaluation scales.

**Data Collection and Ethical Considerations**: Before conducting the survey, the researchers obtained informed consent from each learner, providing a comprehensive explanation of the study's objectives and survey methods. The researchers were physically present in the amphitheater during data collection to maintain a personal connection with the participants. A clear understanding of the study's expectations was provided to all participants, with an emphasis on the absence of right or wrong answers in the questionnaires. Participants were encouraged to respond truthfully and openly, as the accuracy of the results relied on their sincere responses.

Participants were made aware of the research's purpose and how to answer the questions effectively. The data collection process involved learners completing the questionnaires in a specific order: first, the social support questionnaire was administered, followed by the academic enthusiasm, academic vitality, and self-efficacy beliefs surveys. Participants were given ample time to provide thoughtful and comprehensive answers, and no time constraints were imposed.

Ethical considerations were paramount throughout the research. All participants were informed that their participation was entirely voluntary, and they were assured that their information would be kept confidential. The researchers diligently ensured that participants fully understood the study, freely chose to participate, and felt comfortable expressing their viewpoints.

### 3.5 Data analysis

The data collected were analyzed using SPSS 25 and Amos 24 software, encompassing two key sections: descriptive statistics and inferential statistics. In the descriptive statistics section, central indicators and the dispersion of research variables were presented to provide a comprehensive overview of the data.

## 4 Results

### 4.1 Reliability and validity of the questionnaire

#### 4.1.1 Questionnaires

**Assessment of Questionnaire Reliability:** To evaluate the reliability of research questionnaires, researchers computed Cronbach's alpha coefficients. The results revealed high internal consistency, with Cronbach's alpha values of 0.916 for academic enthusiasm, 0.951 for social support, 0.861 for academic vitality, and 0.939 for self-efficacy beliefs. These findings affirm the reliability of our research variables.

**Data Collection Procedure:** Our study engaged 650 participants from Zanjan University. We subsequently selected 242 English learners (representing both genders) aged 18 to 55 for further investigation. The only exclusion criterion was the participants' unwillingness to partake in the intervention.

Data collection took place in February 2023, beginning with the registration of interested individuals at Zanjan University. Subsequently, we administered the Online Questionnaire for English Proficiency Test (OQPT) through an online survey platform, distributing it to each participant via Telegram. Those at the upper intermediate level or above were included in the study.

Upon consultation with the university's vice president, we secured an amphitheater for our research activities. On February 15th, all participants gathered in the amphitheater, where we provided them with consent forms and questionnaires. Participants were given ample time to complete the questionnaires. As a token of appreciation, all participants received a commemorative gift before departing. Before data collection, participants were briefed on the research objectives, emphasizing the voluntary nature of participation and the confidentiality of their responses.

The questionnaires were designed to explore English Language Learners (ELLs) perceptions of social support, academic enthusiasm, academic vitality, and self-efficacy beliefs. Participants received clear instructions for completing the questionnaires, with teachers available to address any inquiries. The researchers personally oversaw data collection at the institutes, ensuring a smooth process and immediate response to any participant queries.

After data collection, the gathered data underwent rigorous statistical analysis to extract meaningful insights and conclusions.

### 4.2 Descriptive findings

The demographic statistics of the variables are presented in Table 1:

**Table 1:** Frequency distribution and age percentage of respondents.

	Category	Frequency	Percent	Cumulative Percent
Age	18–25 years	130	53.7	53.7
	26–30 years	96	39.7	93.4
	35–45 years	16	6.6	100.0
Gender	Male	116	47.9	47.9
	Female	126	52.1	100.0

The results of Table 1 showed that the group of 18–25 years and the group of 26–30 years have the highest frequency and the group of more than 30 years has the lowest frequency. In terms of gender, females were the most abundant with 52.1 %.

The results of the central indices and dispersion of the variables are presented in Table 2:

**Table 2:** Central indicators and dispersion of research variables.

	N	Mean	Std. Deviation	Minimum	Maximum	Skewness	Kurtosis
Academic enthusiasm	242	51.963	10.980	23	70	−0.282	−0.468
Academic vitality	242	30.360	7.043	11	45	−0.337	0.164
Social support	242	92.715	17.337	50	124	−0.309	−0.348
Self-efficacy beliefs	242	56.074	14.392	20	81	−0.507	0.054

The results of descriptive statistics in Table 2 show:

The mean ± standard deviation of the variables of academic enthusiasm is equal to 51.963 ± 10.980, academic vitality equals 30.360 ± 7.043, social support equals 17.337 ± 92.715, and self-efficacy beliefs are equal to 56.074 14.392. Also, the values of skewness and kurtosis are in the range (of −2 and 2), which shows that the data distribution is almost normal.

### 4.3 Inferential statistics

Presuppositions of using the method of structural equations.

Default 1: Kolmogorov-Smirnov test.

To check the normality of the research variables, the Kolmogorov-Smirnov normality test is used, the results of which are as follows:

Based on the results of Table 3, it was observed that the P-value is higher than 0.05 and the assumption of normality of the data is accepted.

**Table 3:** Checking the normality of the variables from the Kolmogorov-Smirnov test.

	Test statistic (K–S)	P-value
Academic Enthusiasm	0.042	0.200
Academic Vitality	0.047	0.187
Social Support	0.042	0.200
Self-efficacy Beliefs	0.051	0.159

### 4.4 Research hypotheses

**Fig. 1:**
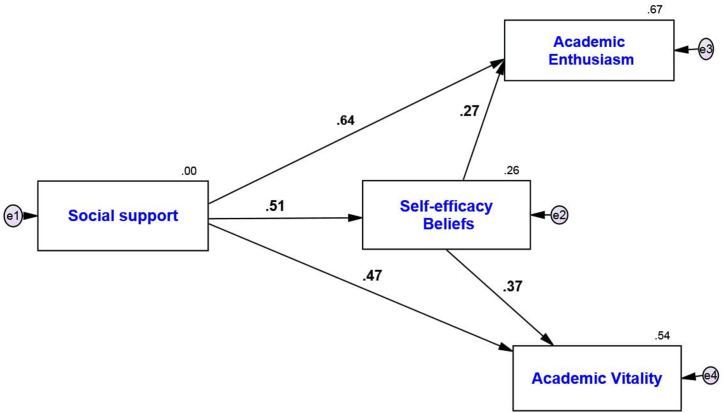
Model fit in standard estimation mode.

**Fig. 2:**
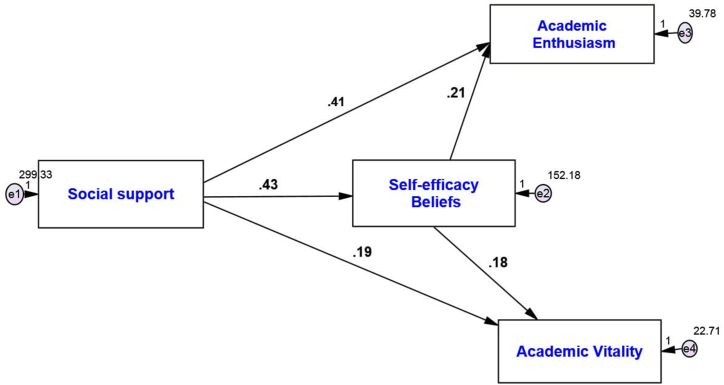
Model fit in non-standard estimation mode.

In the following, the path analysis method with Amos24 software was used to check the relationship between research variables. The research model is as follows (Fig. 1, Fig. 2):

According to the output of the software, the calculated value of 2χ is less than 3 compared to its degree of freedom, i.e., 2 times 2.872. The low level of this index indicates a small difference between the conceptual model and the observed research data. The value of the Root Mean Squared Error of Approximation (RMSEA) is equal to 0.069 and less than 0.08. The indices of Goodness of Fit Index (GFI), Normed Fit Index (NFI), Incremental Fit Index (IFI), Relative Fit Index (RFI), and Comparative Fit Index (CFI) indices are respectively equal to 0.935, 0.936, 0.938, 0.918, and 0.937, which indicate a high fit. In Table 4, path coefficients and t-values were examined for the relationships among the researched variables:

Hypothesis No. 1 Social support has a positive and significant relationship with academic enthusiasm in university students.

**Table 4:** Examination of relationships among research variables.

	Path coefficients in estimation mode standard	t-value	p-value	Condition
Social Support→Academic Enthusiasm	0.644	14.921	0.001**	Accept
Social Support →Academic Vitality	0.473	9.304	0.001**	Accept
Self-efficacy Beliefs → Academic Enthusiasm	0.273	6.314	0.001**	Accept
Self-efficacy Beliefs → Academic Vitality	0.370	7.282	0.001**	Accept
Social Support→ Self-efficacy Beliefs → Academic Enthusiasm	0.139	6.622	0.001**	Accept
Social support→ Self-efficacy beliefs → Academic vitality	0.189	8.882	0.001**	Accept

** ** significant at the 0.01 level (p < 0.01).

The results of path analysis in Fig. 2 and Table 4 show:

The standard coefficient between social support and academic enthusiasm is equal to 0.644, and according to the absolute value of the *t*-test statistic, which is equal to 14.921 and greater than 1.96, it can be concluded with a 99 % probability that social support has a positive and significant effect on academic enthusiasm (p-value = 0/001; β = 0.644) in other words, for an increase of one unit of social support, academic enthusiasm increases by 0.644 units.

Hypothesis No. 2 Social support has a positive and significant relationship with academic vitality in university students.

The results of path analysis in Fig. 2 and Table 4 show:

The standard coefficient between social support and academic vitality is equal to 0.473, and according to the absolute value of the *t*-test statistic, which is equal to 9.304 and greater than 1.96, it can be concluded with a probability of 99 % that social support has a positive and significant effect on academic vitality (p-value = 0/001; β = 0.473) in other words, for an increase of one unit of social support, academic vitality increases by 0.473 units.

Hypothesis No. 3 Self-efficacy beliefs have a positive and significant relationship with academic enthusiasm in university learners.

The results of path analysis in Fig. 2 and Table 4 show:

The standard coefficient between self-efficacy beliefs and academic enthusiasm is equal to 0.273, and according to the absolute value of the *t*-test statistic, which is equal to 6.314 and greater than 1.96, it can be concluded with a 99 % probability that self-efficacy beliefs have a positive and significant effect on academic enthusiasm (p-value = 0/001; β = 0.273) in other words, for one unit increase in self-efficacy beliefs, academic enthusiasm increases by 0.273 units.

Hypothesis No. 4 Self-efficacy beliefs have a positive and significant relationship with academic vitality in university language learners.

The results of path analysis in Fig. 2 and Table 4 show:

The standard coefficient between self-efficacy beliefs and academic vitality is equal to 0.370, and according to the absolute value of the *t*-test statistic, which is equal to 7.282 and greater than 1.96, it can be concluded with a 99 % probability that self-efficacy beliefs have a positive and significant effect on academic vitality (p-value = 0/001; β = 0.370) in other words, for an increase of one unit of self-efficacy beliefs, academic vitality increases by 0.370 units.

Hypothesis No. 5 Social support has a positive and significant relationship with academic enthusiasm and the mediating role of self-efficacy beliefs in university students.

The results of the Sobel test in Table 4 show:

The standard coefficient between social support and academic enthusiasm with the mediating role of self-efficacy beliefs is equal to 0.139 and according to the absolute value of the *t*-test statistic which is equal to 6.622 and greater than 1.96, it can be concluded with a 99 % probability that social support has a positive and significant effect on academic enthusiasm has a mediating role of self-efficacy beliefs (p-value = 0.001; β = 0.139), in other words, for an increase of one unit of social support, academic enthusiasm with a mediating role of self-efficacy beliefs increases by 0.139 units.

Hypothesis No. 6 Social support has a positive and significant relationship with academic vitality and the mediating role of self-efficacy beliefs in university students.

The results of this hypothesis are presented in the following table:

The results of the Sobel test in Table 4 show:

The standard coefficient between social support and academic vitality with the mediating role of self-efficacy beliefs is equal to 0.189, and according to the absolute value of the *t*-test statistic, which is equal to 8.882 and greater than 1.96, it can be concluded with a 99 % probability that social support has a positive and significant effect on academic vitality has a mediating role of self-efficacy beliefs (p-value = 0.001; β = 0.189), in other words, for an increase of one unit of social support, academic vitality with a mediating role of self-efficacy beliefs increases by 0.189 units.

## 5 Discussion

1. There is a significant relationship between social support and academic enthusiasm among EFL university language learners.

The hypothesis states that social support significantly affects academic enthusiasm among EFL university language learners. To evaluate this hypothesis, it is important to discuss the existing research and provide an analysis of the findings.

Various studies have investigated the relationship between social support and academic enthusiasm among university language learners, supporting the hypothesis [20,50]. Social support, which encompasses emotional, instrumental, and informational assistance from social networks, has been found to have a positive impact on academic enthusiasm [35]. When language learners perceive higher levels of social support from peers, instructors, and family members, they exhibit greater passion and interest in their academic pursuits **(**Chouhy et al., 2020 [2]; Falcon et al. [3])**.**

One possible explanation for this relationship is the role of social support in fostering a sense of belonging and validation. Language learners who feel supported by their social networks experience a greater sense of belongingness within the academic environment, which, in turn, enhances their enthusiasm for learning (Wang & Sheikh [9]). The presence of supportive individuals who provide encouragement, guidance, and positive feedback can fuel students' motivation and ignite their passion for academic achievements.

10.13039/100014337Furthermore, social support can positively influence cognitive and emotional behaviors related to academic engagement. When language learners receive social support, they are more likely to actively engage in academic activities, participate in discussions, seek help when needed, and demonstrate higher levels of motivation and perseverance [35]. The availability of supportive resources and relationships provides language learners with the necessary encouragement and confidence to approach their academic challenges with enthusiasm and a growth mindset.

Additionally, social support can alleviate stress and reduce feelings of isolation, both of which can hinder academic enthusiasm. When language learners have a strong support system, they experience less anxiety and feel more secure in their academic pursuits, allowing them to fully immerse themselves in their studies and maintain their enthusiasm (Wang & Sheikh[9]).

It is important to note that the relationship between social support and academic enthusiasm may be influenced by various contextual factors, such as cultural differences, individual characteristics, and the specific nature of the support received. Different cultural backgrounds may shape the expectations and preferences for social support, thus influencing its impact on academic enthusiasm [35]. Moreover, individual differences, such as personality traits and previous experiences, can modulate the extent to which social support affects academic enthusiasm.

Regarding the second hypothesis: There is a significant relationship between social support and academic vitality among EFL university language learners.

The hypothesis suggests that social support significantly affects academic vitality among university language learners. To discuss this hypothesis, it is important to examine relevant research and provide an analysis of the findings.

Several studies have explored the relationship between social support and academic vitality, providing evidence to support the hypothesis ([38]; Brackett et al.[51] [42]). Social support, encompassing emotional, instrumental, and informational assistance from social networks, has been found to have a positive impact on academic vitality (Dehghanizadeh et al. [7]). When language learners perceive higher levels of social support from their peers, instructors, and family members, it enhances their overall academic well-being and vitality.

One explanation for this relationship lies in the role of social support in promoting positive psychological and emotional states. Language learners who have a strong support system are more likely to experience positive emotions, such as enthusiasm, engagement, and a sense of purpose, in their academic pursuits (Dehghanizadeh et al.[7]). Social support provides individuals with the necessary encouragement, motivation, and validation, which can contribute to their overall well-being and vitality within the academic context.

10.13039/100014337Furthermore, social support plays a crucial role in mitigating stress and reducing feelings of isolation, both of which can negatively impact academic vitality. When language learners feel supported by their social networks, they are better equipped to cope with academic challenges, handle setbacks, and maintain a sense of connectedness, thereby promoting their academic vitality (Wang & Sheikh [9]). The availability of supportive relationships and resources provides language learners with a sense of security and belonging, which are fundamental for their overall well-being and academic success.

Additionally, social support can enhance academic vitality by fostering a supportive learning environment. Language learners who receive support from their peers and instructors are more likely to actively engage in collaborative learning, seek help when needed, and participate in academic activities with enthusiasm (Wang & Sheikh, [9]). The presence of a supportive network encourages language learners to fully invest themselves in their academic pursuits, leading to increased vitality and a more positive academic experience.

However, it is important to acknowledge that the relationship between social support and academic vitality may be influenced by various contextual factors and individual differences. Cultural variations, for instance, can shape the expectations and availability of social support, potentially influencing its impact on academic vitality (Dehghanizadeh et al. [7]). Moreover, individual characteristics, such as personality traits and previous experiences, can modulate the extent to which social support affects academic vitality.

### 5.1 Regarding the third hypothesis

There is a significant relationship between self-efficacy beliefs and academic enthusiasm of EFL university language learners. To address this question, it is necessary to examine relevant research and provide an analysis of the findings.

Various studies have investigated the relationship between academic enthusiasm and self-efficacy beliefs, providing evidence to suggest a significant relationship between the two constructs. Academic enthusiasm refers to the passion, interest, and excitement that individuals experience in their academic pursuits, while self-efficacy beliefs reflect individuals' perceptions of their capabilities to complete tasks and achieve desired outcomes (Sayadi & Soleimani [52]).

Research has consistently shown that higher levels of self-efficacy beliefs are associated with increased academic enthusiasm among university language learners (Zimmerman et al.[14]). When language learners have strong beliefs in their abilities to perform well academically, they are more likely to approach their language learning tasks with enthusiasm and engage in proactive learning behaviors. This is because individuals with high self-efficacy beliefs perceive academic challenges as opportunities for growth and view setbacks as temporary obstacles that can be overcome with effort and effective strategies [11].

Self-efficacy beliefs play a crucial role in shaping individuals' motivation, goal-setting, and perseverance in the face of difficulties. Language learners who possess high self-efficacy beliefs tend to set challenging goals, exert more effort, and demonstrate greater persistence in their language-learning endeavors (Zimmerman et al., 1992). These proactive behaviors contribute to the development of academic enthusiasm as individuals feel a sense of accomplishment and fulfillment when they make progress toward their goals.

Furthermore, self-efficacy beliefs influence individuals' emotional states and attitudes towards learning. Language learners with high self-efficacy beliefs are more likely to experience positive emotions such as excitement, interest, and enjoyment in their language learning activities (Zimmerman et al.[14]). They approach challenges with a sense of confidence and view difficulties as opportunities to enhance their skills and knowledge, fostering academic enthusiasm.

It is important to note that the relationship between academic enthusiasm and self-efficacy beliefs can be bidirectional. While high self-efficacy beliefs contribute to increased academic enthusiasm, the experience of academic enthusiasm can also reinforce individuals' self-efficacy beliefs. When language learners feel enthusiastic and passionate about their academic pursuits, they gain a sense of competence and mastery, reinforcing their beliefs in their capabilities.

However, it is crucial to consider individual differences and contextual factors that may influence the relationship between academic enthusiasm and self-efficacy beliefs. Factors such as prior academic achievement, learning experiences, and external support systems can influence the development and maintenance of self-efficacy beliefs, which in turn impact academic enthusiasm (Zimmerman et al. [14]). Additionally, cultural and contextual factors may shape individuals' beliefs and attitudes toward academic enthusiasm and self-efficacy.

### 5.2 Regarding the fourth hypothesis

There is a significant relationship between self-efficacy beliefs and the academic vitality of EFL university language learners.

The question examines the potentially significant relationship between academic vitality and self-efficacy beliefs among university language learners. To address this question, it is important to review relevant literature and provide an analysis of the findings.

Academic vitality refers to the overall energy, engagement, and motivation that individuals experience in their academic pursuits. On the other hand, self-efficacy beliefs reflect individuals' perceptions of their capabilities to successfully perform tasks and achieve desired outcomes (Laouni, 2023). Exploring the relationship between academic vitality and self-efficacy beliefs can shed light on the motivational factors influencing language learners' academic engagement and persistence [53].

Studies have indicated a significant relationship between academic vitality and self-efficacy beliefs among university language learners. Higher levels of self-efficacy beliefs are positively associated with increased academic vitality [54]. When language learners possess strong beliefs in their abilities to succeed academically, they are more likely to approach their language learning tasks with enthusiasm, energy, and motivation.

Self-efficacy beliefs play a crucial role in shaping individuals' engagement and persistence in academic activities. Language learners with high self-efficacy beliefs tend to set challenging goals, demonstrate greater effort, and exhibit higher levels of resilience when faced with obstacles or setbacks (Bandura [55]). These proactive behaviors contribute to the development of academic vitality as individuals experience a sense of purpose, drive, and fulfillment in their language-learning endeavors.

Furthermore, self-efficacy beliefs influence individuals' emotional experiences and attitudes toward learning. Language learners with high self-efficacy beliefs are more likely to experience positive emotions such as excitement, interest, and enjoyment in their academic pursuits (Bandura ). They perceive challenges as opportunities for growth and view their academic activities as meaningful and personally relevant, which contributes to the enhancement of academic vitality.

However, it is crucial to consider individual differences and contextual factors that may influence the relationship between academic vitality and self-efficacy beliefs. Factors such as prior academic achievement, learning experiences, and support systems can impact the development and maintenance of self-efficacy beliefs, which in turn affect academic vitality (Bandura [55]). Additionally, cultural and contextual factors may shape individuals' attitudes and experiences related to academic vitality and self-efficacy.

### 5.3 Regarding the fifth hypothesis

Social support has a significant on academic enthusiasm with the mediating role of self-efficacy beliefs in EFL university language learners.

The hypothesis explores whether social support significantly affects academic enthusiasm by assuming the mediating role of self-efficacy among university language learners. To address this question, it is important to review relevant literature and provide an analysis of the findings.

Social support refers to the resources, assistance, and emotional connections individuals receive from their social networks, such as family, friends, and peers. Academic enthusiasm reflects individuals' passion, interest, and excitement towards their academic pursuits. Self-efficacy, on the other hand, refers to individuals' beliefs in their capabilities to successfully perform tasks and achieve desired outcomes (Wu et al.[34]**;** [13]).

Research studies have examined the relationship between social support, academic enthusiasm, and self-efficacy in university language learners. The findings suggest that social support can significantly affect academic enthusiasm, both directly and indirectly through its impact on self-efficacy.

Direct Relationship: Social support plays a crucial role in fostering a positive academic environment and enhancing students' motivation and engagement. When university language learners perceive higher levels of social support, such as encouragement, guidance, and assistance from their social networks, they are more likely to experience increased academic enthusiasm. Social support provides students with a sense of belonging, validation, and encouragement, which fuels their passion and interest in their language learning journey.

Mediating Role of Self-efficacy: Self-efficacy beliefs act as a mediator in the relationship between social support and academic enthusiasm. Social support can influence individuals' self-efficacy beliefs by providing them with the necessary resources, feedback, and encouragement to overcome challenges and succeed academically [8]. When language learners receive social support, it enhances their confidence, belief in their abilities, and perceptions of competence, which in turn boosts their academic enthusiasm. Self-efficacy beliefs serve as a pathway through which social support influences academic enthusiasm.

Furthermore, self-efficacy beliefs play a crucial role in shaping individuals' perceptions and interpretations of social support. Language learners with high self-efficacy beliefs are more likely to seek and utilize social support effectively. They view social support as a valuable resource that can enhance their language learning outcomes and fuel their academic enthusiasm. On the other hand, individuals with low self-efficacy beliefs may be less likely to seek or fully benefit from social support, which can hinder their academic enthusiasm.

### 5.4 Regarding the sixth hypothesis

Social support has a significant on academic vitality with the mediating role of self-efficacy beliefs in university language learners.

The hypothesis explores how social support significantly affects academic vitality by assuming the mediating role of self-efficacy beliefs in university language learners. To address this hypothesis, it is important to review relevant literature and provide an analysis of the findings.

Social support refers to the resources, assistance, and emotional connections individuals receive from their social networks, such as family, friends, and peers. Academic vitality reflects individuals' sense of energy, engagement, and well-being in their academic pursuits. Self-efficacy beliefs, on the other hand, refer to individuals' beliefs in their capabilities to successfully perform tasks and achieve desired outcomes [37].

Research studies have examined the relationship between social support, academic vitality, and self-efficacy in university language learners. The findings suggest that social support can significantly affect academic vitality, both directly and indirectly through its impact on self-efficacy [41].

Direct Relationship: Social support plays a crucial role in promoting a positive academic environment and enhancing students' well-being and engagement. When university language learners perceive higher levels of social support, such as emotional support, instrumental support, and informational support from their social networks, they are more likely to experience increased academic vitality. Social support provides individuals with a sense of belonging, encouragement, and access to resources, which contribute to their overall academic well-being and vitality.

Mediating Role of Self-efficacy: Self-efficacy beliefs act as a mediator in the relationship between social support and academic vitality. Social support can influence individuals' self-efficacy beliefs by providing them with the necessary support, feedback, and resources to overcome challenges and thrive academically. When language learners receive social support, it enhances their confidence, belief in their abilities, and perceptions of competence, which in turn boosts their academic vitality. Self-efficacy beliefs serve as a pathway through which social support influences academic vitality.

However, it is important to consider that the relationship between social support, self-efficacy, and academic vitality can be influenced by individual differences and contextual factors. Factors such as the quality of social relationships, cultural norms, and the availability of support systems can impact the effectiveness of social support in promoting self-efficacy and academic vitality (Bandura [55]). Additionally, personal characteristics, previous experiences, and other academic-related factors may shape individuals' perceptions and utilization of social support.

## 6 Conclusion

This study delved into the impact of social support on academic enthusiasm and academic vitality among ELLs, considering the mediating role of self-efficacy beliefs. The findings underscore the substantial influence of social support on both academic enthusiasm and academic vitality, with self-efficacy beliefs emerging as a pivotal mediator in this intricate relationship.

The study's results suggest that social support plays a pivotal role in augmenting academic enthusiasm and academic vitality among ELLs. As ELLs perceive heightened levels of social support, they cultivate a positive mindset characterized by emotional, instrumental, and informational aid from peers, mentors, and other members of the academic community. This supportive atmosphere fosters the development of self-efficacy beliefs, which, in turn, significantly enhance academic enthusiasm and academic vitality.

Self-efficacy beliefs serve as the conduit through which social support influences academic enthusiasm and academic vitality. ELLs who receive substantial social support are more likely to nurture higher self-efficacy beliefs, as their socio-emotional needs are met, and they feel valued, acknowledged, and bolstered in their academic journey. These amplified self-efficacy beliefs contribute to elevated levels of academic enthusiasm and academic vitality among ELLs, as they exude enthusiasm, engagement, and vitality in their pursuit of knowledge.

In conclusion, social support is a linchpin in shaping academic enthusiasm and vitality among ELLs, with self-efficacy beliefs serving as the bridge between them. By comprehending and addressing the factors that contribute to social support and self-efficacy beliefs, educational institutions can cultivate an enriching learning environment that fosters enthusiasm and vitality among ELLs, ultimately leading to improved performance, heightened self-efficacy beliefs, and enhanced learning outcomes.

### 6.1 Implication

This study's findings have several ramifications. First, learners may be more likely to find ways to increase levels of academic enthusiasm and academic vitality which can be implemented through the utilization of social support by assuming the mediating role of self-efficacy beliefs. As a result, via strong connections based on social support, self-efficacy beliefs, academic enthusiasm, and academic vitality, learners sound to be better able to handle hardships in learning and to comprehend better. In addition to what was said above, learners' level of learning and perceiving the main concepts will be increased significantly due to the main positive impacts of social support and self-efficacy beliefs on academic enthusiasm and academic vitality. This implies that the administration of constructive methods that support learners can help increase their enthusiasm and vitality toward learning. Moreover, when considering the findings from the self-efficacy beliefs facets, it is recommended that the Ministry of Education examine openly how to improve learners' enthusiasm and vitality by providing sufficient facilities along with advancement, contingent rewards, and working conditions.

### 6.2 Suggestions

1. Conducting research in non-student statistical communities can examine the appropriateness of the presented relationship.
2. Considering that academic enthusiasm and academic vitality are two simple variables and a general and abstract concept, it is suggested that future researchers explore the concept of academic enthusiasm and vitality through numerous and diverse interviews; To measure enthusiasm and academic vitality more accurately.
3. Since the self-efficacy perceived by a person can be somewhat different from the actual performance of people, it is suggested to use methods that include observing people in social situations to measure self-efficacy in future research.
4. According to the findings of the current research, it can be suggested to increase the enthusiasm and academic vitality of the graduates, to increase the social support from the family and the community, which increases self-efficacy beliefs in people, so that people with confidence in their abilities, have higher motivation in the field of education.

This study used social support to look at how it affected the academic enthusiasm, academic vitality, and self-efficacy beliefs of EFL learners. Future studies might approach the issue differently. Only surveys allowed researchers to examine how social support affected EFL learners' academic enthusiasm, academic vitality, and self-efficacy beliefs. In subsequent studies, researchers may employ strategies like triangulation, observation, journaling, and interviews to obtain more generalizable data. In the latest study, each variable's impact on social support was investigated independently. The relationship between social support and other variables like academic enthusiasm, academic vitality, and self-efficacy beliefs could be examined using models. Future studies may examine the problem in terms of additional demographic variables such as gender, age, and linguistic ability. The investigation was conducted in centers for language instruction. It ought to be replicated in a variety of contexts, including academic, public, and commercial organizations, where students may see the surveys from various angles depending on their needs and circumstances.

## Availability of data and materials

The data will be available upon request from the corresponding author.

## Informed consent

Written informed consent was obtained from all subjects before the study. There is no ethical or conflict of interest in this research. All the participants filled out consent forms.

## Ethics approval

**Ethics approval:** Approval ID: Research Ethics Committees of Islamic Azad University-Zanjan Branch: IR.IAU.Z.REC.1402.079.

## CRediT authorship contribution statement

**Qin Luo:** Writing – review & editing, Validation, Supervision, Methodology, Formal analysis. **Roya Ahmadi:** Resources, Investigation. **Siros Izadpanah:** Supervision, Methodology, Conceptualization.

## Declaration of competing interest

The authors declare that they have no known competing financial interests or personal relationships that could have appeared to influence the work reported in this paper.
