# Supplementary material for: Exploring the mediating role of self-efficacy beliefs among EFL university language learners: The relationship of social support with academic enthusiasm and academic vitality
Source: Heliyon. 2024 Jun 18;10(12):e33253. doi: 10.1016/j.heliyon.2024.e33253 (PMC11252873; doi:10.1016/j.heliyon.2024.e33253)
Supplement: Multimedia component 1 [file mmc1.docx]

***APPENDICES***

Appendix A (Social Support Questionnaire)

A – Very much

B – Much

C – Medium

D – Little

E – Very little

| E | D | C | B | A |  |
| --- | --- | --- | --- | --- | --- |
|  |  |  |  |  | 1. My friends respect me. |
|  |  |  |  |  | 2. My family takes great care of me. |
|  |  |  |  |  | 3. Other people don't care about me. |
|  |  |  |  |  | 4. My family respects me a lot. |
|  |  |  |  |  | 5. I am very popular. |
|  |  |  |  |  | 6. I can rely on my friends. |
|  |  |  |  |  | 7. I am completely admired by my family. |
|  |  |  |  |  | 8. Others pay attention to me. |
|  |  |  |  |  | 9. My family loves me sincerely. |
|  |  |  |  |  | 10. My friends do not care about my happiness and success. |
|  |  |  |  |  | 11. My family members rely on me. |
|  |  |  |  |  | 12. I have a lot of confidence. |
|  |  |  |  |  | 13. I cannot rely on the support and assistance of my family members. |
|  |  |  |  |  | 14. People admire and praise me. |
|  |  |  |  |  | 15. I feel very fond of my friends. |
|  |  |  |  |  | 16. My friends like me. |
|  |  |  |  |  | 17. Others value me. |
|  |  |  |  |  | 18. My family respects me. |
|  |  |  |  |  | 19. My relationship with my friends is very important to me. |
|  |  |  |  |  | 20. I want an attachment to others. |
|  |  |  |  |  | 21. If I died tomorrow, few people would miss me. |
|  |  |  |  |  | 22. I feel that I am not close to my family members. |
|  |  |  |  |  | 23. My friends and I help each other in difficult times. |

**پرسشنامه حمایت اجتماعی**

| گزینه ها | خیلی زیاد | زیاد | متوسط | کم | خیلی کم |
| --- | --- | --- | --- | --- | --- |
| 1.      دوستانم به من احترام می گذارند. |  |  |  |  |  |
| 2.      خانواده ام مرا مورد مراقبت بسیار قرار می دهند. |  |  |  |  |  |
| 3.      دیگران برای من اهمیتی قائل نیستند. |  |  |  |  |  |
| 4.      خانواده ام برای من حرمت فراوانی قائل هستند. |  |  |  |  |  |
| 5.      من خیلی محبوب هستم. |  |  |  |  |  |
| 6.      من میتوانم به دوستانم تکیه کنم |  |  |  |  |  |
| 7.      کاملا مورد تحسین خانواده ام هستم. |  |  |  |  |  |
| 8.      دیگران به من اعتنا می کنند. |  |  |  |  |  |
| 9.      خانواده ام صمیمانه به من عشق می ورزند. |  |  |  |  |  |
| 10.   دوستانم نسبت به سعادت و موفقیت من اعتنایی ندارند. |  |  |  |  |  |
| 11.   اعضاء خانواده ام به من متکی هستند. |  |  |  |  |  |
| 12.   من از اعتماد زیادی برخوردار هستم. |  |  |  |  |  |
| 13.   من نمی توانم به حمایت و مساعدت افراد خانواده ام متکی باشم. |  |  |  |  |  |
| 14.   مردم مرا تحسین و ستایش می کنند. |  |  |  |  |  |
| 15.   من احساس می کنم به دوستانم علاقه زیادی دارم. |  |  |  |  |  |
| 16.   دوستانم هوای مرا دارند. |  |  |  |  |  |
| 17.   دیگران برای من ارزش قائل هستند. |  |  |  |  |  |
| 18.   خانواده ام واقعاً به من احترام می گذارند. |  |  |  |  |  |
| 19.   رابطه من با دوستانم برایم خیلی مهم است. |  |  |  |  |  |
| 20.   من خواهان دلبستگی به دیگران هستم. |  |  |  |  |  |
| 21.   اگر فردا بمیرم، افراد کمی برای من احساس دلتنگی می کنند. |  |  |  |  |  |
| 22.   احساس میکنم با اعضای خانواده ام صمیمی نیستم. |  |  |  |  |  |
| 23.   من و دوستانم در روزهای دشوار و سختی به درد همدیگر می خوریم. |  |  |  |  |  |

Appendix B (Academic Enthusiasm Questionnaire)

A – Very little

B – Little

C – Medium

D – Much

E – Very much

| E | D | C | B | A |  |
| --- | --- | --- | --- | --- | --- |
|  |  |  |  |  | 1. I pay attention in class. |
|  |  |  |  |  | 2. I just pretend to be active when I'm in class. |
|  |  |  |  |  | 3. I follow the school rules. |
|  |  |  |  |  | 4. I get into trouble at school. |
|  |  |  |  |  | 5. I feel happy at school. |
|  |  |  |  |  | 6. I am bored at school. |
|  |  |  |  |  | 7. I get excited about school activities. |
|  |  |  |  |  | 8. I like to stay at school. |
|  |  |  |  |  | 9. I am interested in school activities. |
|  |  |  |  |  | 10. The classroom is an interesting place to stay. |
|  |  |  |  |  | 11. When I read a book, I ask myself questions to make sure I understand the topic. |
|  |  |  |  |  | 12. I study at home even when I don't have an exam. |
|  |  |  |  |  | 13. I try to watch TV programs about our activities at school. |
|  |  |  |  |  | 14. I definitely check my homework to fix the problems. |
|  |  |  |  |  | 15. I read extracurricular books to learn more. |

**پرسشنامه اشتیاق تحصیلی**

| **عبارات** | **خیلی کم** | **کم** | **متوسط** | **زیاد** | **خیلی زیاد** |
| --- | --- | --- | --- | --- | --- |
| 1.من در کلاس توجه می کنم. |  |  |  |  |  |
| 2. وقتی سر کلاس هستم فقط تظاهر می کنم که فعال هستم |  |  |  |  |  |
| 3. من از قوانین مدرسه تبعیت می کنم. |  |  |  |  |  |
| 4. من در مدرسه دچار مشکل می شوم. |  |  |  |  |  |
| 5. من در مدرسه احساس خوشنودی می کنم**.** |  |  |  |  |  |
| 6. من در مدرسه حوصله ام سر می رود**.** |  |  |  |  |  |
| 7. من از فعالیت در مدرسه هیجان زده می شوم. |  |  |  |  |  |
| 8. من دوست دارم در مدرسه بمانم**.** |  |  |  |  |  |
| 9. من علاقه مند به فعالیت در مدرسه هستم. |  |  |  |  |  |
| 10.کلاس درس جای جالبی برای ماندن است. |  |  |  |  |  |
| 11.وقتی کتابی را می خوانم ، از خودم سوال می پرسم تا مطمئن شوم موضوع انرا فهمیده ام. |  |  |  |  |  |
| 12.حتی زمانی که امتحان ندارم در خانه درس می خوانم. |  |  |  |  |  |
| 13.تلاش می کنم برنامه های تلویزیون را که در باره فعالیت های ما در مدرسه را تماشا کنم. |  |  |  |  |  |
| 14.من حتما تکالیف درسی ام را برای رفع اشکال چک می کنم. |  |  |  |  |  |
| 15.من برای یادگیری بیشتر دروس ، کتاب های فوق برنامه مطالعه می کنم . |  |  |  |  |  |

Appendix C (Academic vitality Questionnaire)

Completely disagree Completely agree

1 2 3 4 5 6 7

|  | 1 | 2 | 3 | 4 | 5 | 6 | 7 |
| --- | --- | --- | --- | --- | --- | --- | --- |
| 1. If one day I get a bad grade in school, he still knows that I will have a good and happy day. |  |  |  |  |  |  |  |
| 1. If there are many lessons and I don't have enough time, I know how to cope with the situation and be happy. |  |  |  |  |  |  |  |
| 1. I know that if I get a bad grade, my confidence will still be high. |  |  |  |  |  |  |  |
| 1. If the teacher writes "very bad" under my sheet or notebook, I will be careful not to break my nerves and ruin my day. |  |  |  |  |  |  |  |
| 1. I don't let the stress of studying destroy my joys. |  |  |  |  |  |  |  |
| 1. If I get a bad grade, it doesn't make me unhappy with my friends at school. |  |  |  |  |  |  |  |
| 1. Even on the days when I am not sufficiently prepared for the exam, I am happy with the schoolchildren. |  |  |  |  |  |  |  |
| 1. If I don't have enough time to study; Again, I try to maintain my confidence. |  |  |  |  |  |  |  |
| 1. If the teacher asks me a question and I can't answer, I keep my cool and I'm good-natured after class. |  |  |  |  |  |  |  |

**پرسشنامه سرزندگی تحصیلی**

| ردیف | **عبارات** | **1** | **2** | **3** | **4** | **5** | **6** | **7** |
| --- | --- | --- | --- | --- | --- | --- | --- | --- |
| 1 | اگر روزی در مدرسه نمره ی بدی بگیرم، باز هم می داند که روز خوب و خوشی خواهم داشت. | 1 | 2 | 3 | 4 | 5 | 6 | 7 |
| 2 | اگر درسها زیاد باشند و من وقت کافی نداشته باشم می دانم چطور با شرایط کنار بیایم و خوش هم باشم. | 1 | 2 | 3 | 4 | 5 | 6 | 7 |
| 3 | می دانم که اگر نمره بدی بگیرم، همچنان اعتماد به نفسم بالا خواهد بود. | 1 | 2 | 3 | 4 | 5 | 6 | 7 |
| 4 | لگرمعلم زیر برگه یا دفترم بنویسد: «خیلی بد» مواظب هستم اعصابم خرد نشود و روزم خراب نشود. | 1 | 2 | 3 | 4 | 5 | 6 | 7 |
| 5 | اجازه نمی دهم استرس درس خواندن خوشی هایم را از بین ببرد. | 1 | 2 | 3 | 4 | 5 | 6 | 7 |
| 6 | اگر نمره ی بدی بگیرم، باعث نمی شود با دوستانم در مدرسه خوش نباشم. | 1 | 2 | 3 | 4 | 5 | 6 | 7 |
| 7 | حتی روزهایم که برای امتحان آمادگی کافی ندارم، در کنار بچه های مدرسه خوش هستم. | 1 | 2 | 3 | 4 | 5 | 6 | 7 |
| 8 | اگر وقت کافی برای درس خواندن نداشته باشم؛ باز هم سعی می کنم اعتماد به نفسم را حفظ کنم. | 1 | 2 | 3 | 4 | 5 | 6 | 7 |
| 9 | اگر معلم از من بپرسد و نتوانم جواب دهم، خونسردی خودم را حفظ می کنم و بعد از کلاس هم خوش اخلاقم. | 1 | 2 | 3 | 4 | 5 | 6 | 7 |

Appendix D (Self-efficacy Beliefs Questionnaire)

A – Completely disagree

B – Disagree

C – Disagree a little

D – Agree

E – Completely agree

| E | D | C | B | A |  |
| --- | --- | --- | --- | --- | --- |
|  |  |  |  |  | 1. When I make a plan, I'm sure I can do it. |
|  |  |  |  |  | 2. One of my problems is that when I have to do something, I can't do it. |
|  |  |  |  |  | 3. If I can't do something the first time, I keep trying to do it. |
|  |  |  |  |  | 4. When I set important goals for myself, I rarely achieve them. |
|  |  |  |  |  | 5. I leave it before I do all my work. |
|  |  |  |  |  | 6. I avoid facing problems. |
|  |  |  |  |  | 7. If something seems too complicated, I don't even bother to try it. |
|  |  |  |  |  | 8. When I have to do something inappropriate, I am persistent enough to finish it. |
|  |  |  |  |  | 9. When I decide to do something, I seriously and carefully focus on doing the same thing. |
|  |  |  |  |  | 10. When I try to learn something new, I soon give up if I don't succeed at first. |
|  |  |  |  |  | 11. When unexpected problems happen to me, I do not cope well. |
|  |  |  |  |  | 12. I avoid learning new experiences when I find them difficult. |
|  |  |  |  |  | 13. Failure makes me work harder. |
|  |  |  |  |  | 14. I don't trust my ability to do things. |
|  |  |  |  |  | 15. I rely on myself. |
|  |  |  |  |  | 16. I simply give up. |
|  |  |  |  |  | 17. I can't deal with most of the problems that come to me in life. |

**پرسشنامه باورهای خودکارآمدی**

| **ردیف** | **جملات** | کاملا  مخالفم | مخالفم | حد وسط | موافقم | کاملا  موافقم |
| --- | --- | --- | --- | --- | --- | --- |
| 1 | وقتی طرحی می ریزم، مطمئن هستم می توانم آن را انجام دهم. |  |  |  |  |  |
| 2 | یکی از مشکلات من این است که وقتی می بایست کاری انجام دهم ، نمی توانم از عهده آن برآیم. |  |  |  |  |  |
| 3 | اگر نتوانم کاری را بار اول انجام دهم، به تلاشم برای انجام آن ادامه می دهم. |  |  |  |  |  |
| 4 | وقتی که اهداف مهم برای خود تعیین می کنم ، به ندرت به آن ها دست می یابم. |  |  |  |  |  |
| 5 | قبل از انجام تمام کارهایم آن را رها می کنم. |  |  |  |  |  |
| 6 | از روبه رو شدن با مشکلات ، اجتناب می کنم. |  |  |  |  |  |
| 7 | در صورتی که کاری خیلی پیچیده به نظر برسد حتی زحمت امتحانش را به خود نمی دهم. |  |  |  |  |  |
| 8 | هنگامی که کاری را باید انجام دهم نا مناسب است ، ان قدر پایداری می کنم تا ان را تمام کنم. |  |  |  |  |  |
| 9 | وقتی تصمیم به انجام کاری گرفتم ، به طور جدی و دقیق روی انجام همان کار تمرکز می کنم. |  |  |  |  |  |
| 10 | هنگامی که سعی می کنم چیز جدیدی بیاموزم اگر در ابتدا موفق نشوم به زودی آن را رها می کنم. |  |  |  |  |  |
| 11 | وقتی مشکلات غیرمترقبه ای برایم رخ می دهد ، به خوبی از پس آن برنمی آیم. |  |  |  |  |  |
| 12 | از یادگیری مطالب تجربه های جدید ، هنگامی که به نظرم مشکل بیایند اجتناب می کنم. |  |  |  |  |  |
| 13 | شکست باعث تلاش بیشتر من می شود. |  |  |  |  |  |
| 14 | به توانایی خود برای انجام کارها اعتماد ندارم. |  |  |  |  |  |
| 15 | به خود متکی هستم. |  |  |  |  |  |
| 16 | به سادگی تسلیم می شوم. |  |  |  |  |  |
| 17 | توانایی برخورد با اغلب مشکلاتی را که در زندگی برایم پیش می آید ، ندارم. |  |  |  |  |  |
